# Redefining the oral health workforce: advancing equity and improving global oral health outcomes through inclusive, strategic reform

**DOI:** 10.3389/fdmed.2025.1670715

**Published:** 2025-10-22

**Authors:** Arish Naresh, Roisin McGrath, Rachael England, Kristina Wanyonyi-Kay, Vinod Kumar, Anishma Ram, Zaeni Dahlan, Silvana Bettiol, Penioni Ravunawa, Leenu Maimanuku, Diane Pevreal, Lesley Naidoo, Tan Minh Nguyen

**Affiliations:** ^1^ Department of Community Oral Health Service, Health New Zealand Tairawhiti, Gisborne, New Zealand; ^2^ College of Health and Medicine, University of Tasmania, Hobart, TAS, Australia; ^3^ Melbourne Dental School, the University of Melbourne, Melbourne, VIC, Australia; ^4^ Oral Health Victoria, Melbourne, VIC, Australia; ^5^ Oral Health Association of Australia, Nundah, QLD, Australia; ^6^ Head of Policy and Advocacy, Oral Health Foundation, Rugby, United Kingdom; ^7^ The Healthcare Improvement Studies Institute, University of Cambridge, Cambridge, United Kingdom; ^8^ Fiji Oral Health Association, Suva, Fiji Islands; ^9^ Pasifika Dental Association, Auckland, New Zealand; ^10^ Department of Policy, Indonesian Health Council, Jakarta, Indonesia; ^11^ Ministry of Health and Medical Services, Suva, Fiji Islands; ^12^ School of Oral Health, Fiji National University, Suva, Fiji Islands; ^13^ School of Dentistry, University of KwaZulu-Natal, Durban, South Africa; ^14^ Deakin Health Economics, Institute for Health Transformation, Deakin University, Melbourne, VIC, Australia; ^15^ Health Economics Group, School of Public Health and Preventive Medicine, Monash University, Melbourne, VIC, Australia

**Keywords:** public health dentistry, universal health care, primary health care, scope of practice, cost effectiveness

## 1 Introduction

Oral health is integral to overall health and wellbeing, yet significant disparities in oral health status exist worldwide. The World Health Organization (WHO) Global strategy and action plan on oral health (GOHAP) 2023–2030 focuses on improving health equity through a broad range of strategies (1), including integrating oral health into Universal Health Coverage (UHC) and strengthening the skill-mix and role of the oral health workforce. Historically, dental hygienists and dental therapists have played crucial roles in delivering essential oral healthcare, particularly in underserved areas. Recognizing the critical role oral health practitioners (OHPs), which collectively includes dental hygienists, dental therapists and oral health therapists, can play, and expanding their scopes of practice to remove age restrictions that currently exist in some countries hindering in provision of care (depending on the country context/jurisdiction), enabling them to deliver a broader range of both treatment and preventive focused services, are important steps toward achieving global health equity. For example, enabling greater autonomy for dental hygienists has significant reductions observed indicators on tooth extractions (2). Multiple lines of evidence have demonstrated the need for a strategic transformation in how we deliver oral healthcare globally, from disease-focused treatment models to person-centred and technology-enabled approaches (3).

## 2 Global oral health inequities

Oral diseases are the most common non-communicable diseases. The 2021 Global Burden of Disease Study found that the prevalence of oral diseases increased from 3.5 billion in 2019–3.7 billion in 2021 (4). The key drivers of changes in these estimates are variations in population size, longevity (life expectancy) and disease levels as well as potential that the public health interventions resulting in disruptions to routine dental practice due to COVID-19 pandemic added to the increase in disease prevalence (4, 5). The continued rise in preventable oral diseases demonstrates that existing models of oral health care delivery are ineffective in addressing oral diseases and failing to meet community health needs.

Significant barriers exist to utilizing oral health care at macro (system), meso (organizational), and micro (clinical) levels (6). These barriers can include the maldistribution of the oral health workforce, cost of healthcare, widening inequalities due to disproportionate implementation of technological advances, the exclusion of oral health in primary health care, and the lack of government commitment to tackling oral health inequalities. To meet the demands of UHC, it is estimated that an additional 43 million healthcare workers are needed worldwide; for the oral health workforce, it is suggested that 8.2 dental personnel are required per 10,000 population (7). The most severe health workforce shortages affect low- and middle-income countries, where the burden of disease is higher and the health system is often under-resourced. Addressing the workforce shortages may require the inclusion of non-dental practitioners, such as Indigenous health workers, in oral health care promotion and oral health care provision (8, 9).

## 3 Expanding the oral health workforce

The OHP workforce has demonstrated their critical role in global healthcare systems. For over a century, oral health practitioners have demonstrated effectiveness in delivering high-quality and safe oral healthcare. While their scope of practice varies significantly between (and sometimes within) countries, they are recognized for their provision of safe and cost-effective oral healthcare, and reducing the need for more complex, invasive and costly treatment interventions.

In New Zealand, the School Dental Service, established in 1921, demonstrated the effectiveness of oral health practitioners (initially called “School Dental Nurses” before a title change to dental therapists) in delivering preventive and restorative care for children, and has evolved into the current Community Oral Health Service. In its time, New Zealand was world-leading in providing regular dental care for children. Similar school and community-based dental service delivery models have expanded to the United Kingdom, Australia and many other countries. Expanding their scopes of practice and training dental assistants for advanced practice roles can alleviate oral health workforce shortages and improve access to care with appropriate registration standards to be implemented alongside the proposed expansion.

The 2025 systematic review of 149 randomized control trials highlighted that 81% of studies identified a positive primary outcome from task-sharing and augmented roles (8). In strengthening and designing healthcare systems, task-sharing ought to be designed and implemented as a team-based approach where all staff members are motivated, trained, appropriately remunerated, and have the government and community's support to achieve to the best of their ability.

Previous research has indicated that an optimal oral health workforce skill-mix is at least 70% oral health therapists and 30% dentists in countries that have existing oral health therapist models (10). With changes to community oral health needs requiring preventive and less complex restorative care, greater utilization of oral health practitioners frees dentists' and clinical expertise to focus on complex treatments, enhancing overall productivity, efficiency and interprofessional teamwork. Oral health practitioners are expertly trained in oral health promotion, which can support expanded access to community oral health programs in underserved communities such as school settings, rural and remote areas, and residential aged care facilities, at lower costs than a dentist-centric model.

A multifaceted approach is required to develop the oral health workforce to address the unmet needs of underserved populations. This includes growing the number of oral health practitioners, removing regulatory barriers enabling autonomy in clinical decision making and practice, and expanding their scope through regulated and accredited upskilling to incorporate a broader range of oral health interventions. There should be an increased focus on interprofessional collaborative practice, involving all members of the dental team working with multi-professional healthcare disciplines to achieve improved health outcomes.

The WHO GOHAP 2023–2030 recommends that by 2030, 50% of countries have an operational national health workforce policy, plan or strategy that includes a workforce trained to respond to population oral health needs. The baseline report, released in 2025, highlighted the lack of accurate workforce data, and the challenges in identifying potential data sources around workforce models and task-sharing. This critical gap in global oral health workforce planning and monitoring needs to be urgently addressed. At the country level, the workforce mix should be guided by national data, needs assessments, and health system context, with such data routinely tracked via National Health Workforce Accounts (1, 11).

## 4 Economic benefits of a diversified oral health workforce

Integration of oral health practitioners within dental teams and broader healthcare settings supports healthcare system effectiveness and sustainability. Beyond oral health, OHPs can be involved in areas such as disease surveillance and monitoring, chronic disease management (e.g., diabetes screening, blood pressure checks), and specialized care for vulnerable populations (e.g., people with disability, victim/survivors of domestic and family violence). Importantly, OHPs preventive focus aligns with the WHO's emphasis on enhancing primary health care (PHC) by addressing the root causes and common risk factors of noncommunicable diseases (NCDs), positioning them as valuable members of integrated, people-centred PHC teams. OHPs are skilled, or can be upskilled, in interventions such as smoking cessation support and dietary counselling and can contribute meaningfully to screening for systemic conditions like diabetes and hypertension. Given that best practice for Idental recall visits are typically scheduled every 3, 6, or 12 months based on an individual's risk profile (12), integrating OHPs into primary care systems offers a practical opportunity to monitor and address NCD risk factors regularly, with a patient centred approach thereby supporting the broader goals of Universal Health Coverage (UHC). It is vital that policy makers, health professionals and colleagues within the primary healthcare setting recognise the crucial role of OHPs and the interlinkages between oral health and overall health (13).

## 5 Oral health in universal health coverage

Integrating oral health into UHC is necessary to ensure all people receive timely access to essential oral healthcare without the risk of financial hardship. Countries such as Denmark, Finland, and Sweden utilise a high number of oral health practitioners, thereby, making it possible to include oral health as part of UHC at proportionally lower national government budgets (13). An optimal oral health workforce skill-mix provides the foundation to achieve UHC that includes oral health. Utilising OHPs, whose scope of practice enables them to deliver essential oral healthcare that is safe, effective and cost-effective is the key to achieving this (14–17).

## 6 One health and oral health

The One Health approach recognises the interconnectedness of human, animal, and environmental health. Oral healthcare practices, such as the use of mercury in dental amalgams, are detrimental to the environment. The carbon footprint of dentistry, like any other healthcare sector, is significant and includes energy consumption, waste production, chemical use and travel for staff and patients. Adopting sustainable waste management, energy efficiency, risk-based timely preventive care and accessible essential services which reduce the need for more resource intensive oral health care, aligns with planetary health goals, promoting both individual and environmental wellbeing (18–20).

## 7 Call to action

### 7.1 Aligning with WHO 2030 and one health goals

Strengthening the oral health workforce aligns with the WHO GOHAP 2023–2030 objectives and the broader One Health framework. Collaborative efforts are necessary to achieve these goals and improve global health outcomes.

National oral health policies are required that support task-sharing across multidisciplinary healthcare teams. Furthermore, healthcare systems need to effectively integrate primary care providers with skills and knowledge on oral health promotion to achieve UHC that includes oral health (10). The argument being strongly put forward is that there is no UHC without oral health.

### 7.2 Adequately resourcing the oral health workforce and integration of OHPs into primary care and community health settings, with a focus on interprofessional collaborative practice

The oral health workforce should be supported with standardized digital tools, equipment and pharmaceutical supplies in line with the WHO Essential Medicines List. E.g., fluoride toothpaste, fluoride varnish and silver diamine fluoride. Beyond the traditional clinical environment, OHPs have a crucial role to play in integrating oral healthcare into primary healthcare and community settings, working collaboratively with other health and non-health professionals.

In a time of global oral healthcare workforce shortages, expansion of the OHP workforce presents a pragmatic and economically viable response. OHPs have training in health promotion, prevention and disease detection, and minimally invasive care. They deliver cost-effective services in diverse settings and are often the first point of contact for communities underserved by traditional oral health systems.

### 7.3 Investment in training and expanding workforce capabilities to meet the needs of local populations

Increased funding for education, continuous professional development, and career advancement opportunities for oral health practitioners is essential. Both public and private health sectors must invest in building a robust and competent oral health workforce.

Development of advanced practitioner modular training that may include, for example, prescribing pain relief medicines and/or antibiotics, administering and monitoring nitrous oxide sedation. Legislative updates need to specifically include oral health practitioners for oral healthcare subsided by government and private health insurance.

### 7.4 Establishment of an internationally recognized set of minimum core competencies for OHPs

Establishing an internationally recognised set of minimum core competencies for OHPs ensures common understanding of the oral health practitioner professions inter-professionally and for the general public, providing consistency for clinical education curricula, training and oral healthcare service delivery. A globally accepted scope of practice for each oral health practitioner profession facilitates recognition of relevant qualifications by health workforce regulators, enables oral health workforce mobility and enhances the quality of oral healthcare provided by dental teams. Enabling professional autonomy should be a key feature for the scope of practice of oral health practitioners, which can increase access to preventive dental services and reduce need for complex dental treatment (16).

### 7.5 International Nomenclature

Standardizing oral health practitioner titles and roles across countries reduce confusion and promote clarity in the oral health workforce to health professionals, policy-decision makers, and consumers. A unified nomenclature supports global collaboration and recognition of these health professionals, particularly individuals qualified in dental therapy and dental hygiene that forms the scope of practice of oral health therapists. Australia was the first jurisdiction to recognise the oral health therapist as a protected regulated health practitioner, a recommendation from the 1993 UK Nuffield Foundation report (17). Minimum tertiary education requirements have also shifted from certificate and diploma-based qualifications (1–2 years) to bachelor degrees (3–4 years), driven by changes to community and higher degree education expectations for the oral health practitioner profession.

## 8 Conclusion

Promoting health equity through universal health coverage that includes oral health cannot be achieved without the rapid expansion and utilisation of oral health practitioners within the health workforce. International collaboration efforts are needed to ensure oral health practitioners are appropriately recognised by governments and the public to significantly reduce the global burden of oral disease. Addressing global oral health disparities requires a transformative approach that recognizes and expands the role of oral health practitioners within healthcare systems. By formalizing their status as autonomous, fully practicing professionals, we can significantly improve access to essential oral health services, especially in underserved and marginalized communities. Evidence consistently demonstrates that integrating oral health practitioners into diverse care models enhances patient outcomes, promotes preventive care, increases access, and increases system efficiency. To realize these benefits, concerted efforts must be made to standardize scopes of practice, strengthen workforce expansion models, and ensure sustainable funding. Embracing a collaborative, team-based approach aligned with the principles of universal health coverage is vital for achieving equitable, person-centered oral health care worldwide. Ultimately, such strategic reforms are essential steps toward advancing global oral health equity, planetary Health and sustainable development.
